# Global disparities in access to diagnostics and therapies for advanced non-small cell lung cancer: from discovery to delivery – a review

**DOI:** 10.3389/fonc.2026.1807704

**Published:** 2026-04-01

**Authors:** Lea Ruge, Malte Verheyen, Felix John, Jürgen Wolf

**Affiliations:** University Hospital of Cologne, Department I of Internal Medicine, Center for Integrated Oncology Aachen Bonn Cologne Düsseldorf, Lung Cancer Group Cologne, Cologne, Germany

**Keywords:** non-small cell lung cancer, NSCLC, disparities, access, diagonstics, molecular testing, reimbursement, low- and middle-income countries

## Abstract

Advanced non–small cell lung cancer (NSCLC) has undergone a profound transformation over the past two decades through the integration of molecular diagnostics, targeted therapies, and immunotherapy into clinical practice. Despite these advances, access to modern diagnostics and treatments remains highly uneven across regions and health-care systems, leading to persistent global disparities in diagnostic accuracy, therapeutic options, and patient outcomes. This review explores diagnostic and therapeutic disparities in advanced NSCLC across high-, middle-, and low-income settings, a disease context that is increasingly dependent on timely access to molecularly guided treatment decisions. We describe regional and income-related differences in the availability and implementation of molecular diagnostics and novel systemic therapies, and discuss structural and systemic factors influencing access to innovation, including health-care infrastructure, regulatory environments, and resource constraints. By synthesizing evidence from international guidelines, real-world studies, and global oncology literature, this narrative review highlights how unequal adoption of advances in NSCLC care continues to contribute to outcome differences worldwide and identifies key challenges relevant to future efforts aimed at reducing inequities.

## Introduction

Lung cancer remains the leading cause of cancer-related mortality worldwide, accounting for approximately 1.8 million deaths annually (1). Incidence patterns vary geographically and reflect differences in tobacco exposure, environmental risk factors, and demographic structures (2, 3). Differences in survival might closely be linked to variations in health system capacity and access to modern diagnostics and systemic therapies. Over the past two decades, survival gains have been disproportionately realized in high-income countries (HICs), while many low- and middle-income countries (LICs, LMICs) continue to experience comparatively limited improvements in outcomes (4).

Non-small cell lung cancer (NSCLC), representing approximately 85% of lung cancer cases, has evolved from a histology-based classification toward a molecularly stratified disease. The identification of actionable oncogenic drivers – including *EGFR* mutations, *ALK* rearrangements, *ROS1* fusions, *BRAF* mutations, *MET* exon 14 skipping alterations, *RET* fusions, and *KRAS* variants – has fundamentally reshaped therapeutic algorithms (5, 6). In parallel, immune checkpoint inhibitors, guided by PD-L1 expression, have expanded treatment options across molecular subgroups. Contemporary management therefore requires routine biomarker assessment at diagnosis, a prerequisite that is not uniformly achievable across health systems.

The prevalence of specific driver mutations varies substantially by ethnicity and geographic region. *EGFR* alterations are detected in approximately 30–40% of Asian patients with advanced NSCLC but in 7–18% of patients in Western populations, whereas *KRAS* mutations predominate in Europe and North America (7–9). Elevated *EGFR* mutation frequencies have also been reported in parts of Latin America, while data from the Middle East, North Africa, and sub-Saharan Africa (SSA) remain comparatively limited (10–12). For other therapeutically relevant driver alterations such as *ALK*, *ROS1*, *RET*, *NTRK*, *METex14Skp*, *HER2* and *BRAF*, geographic variation appears less pronounced and generally remains within the single-digit prevalence ranges (9). These molecular differences inform treatment selection but do not inherently account for observed survival disparities. Rather, their clinical relevance depends on the availability of diagnostic infrastructure capable of detecting actionable alterations and the accessibility of corresponding targeted therapies.

The implementation of precision oncology in NSCLC requires coordinated imaging and pathology services, molecular testing platforms – including next-generation sequencing (NGS) – multidisciplinary expertise, sustainable reimbursement mechanisms for high-cost therapies, and regulatory systems that enable timely drug approval and adoption. Variability across these domains influences whether advances in molecularly guided therapy translate into improved patient outcomes. In several regions, limited testing capacity constrains identification of eligible patients; in others, approved therapies remain financially inaccessible or are introduced with substantial delays. Differences in clinical trial infrastructure further affect patient access to innovative treatments.

Although global variation in lung cancer epidemiology and inequities in access to cancer care have been described, these domains are frequently addressed separately. Integrating disparities in diagnostic capacity, biomarker testing, systemic therapy availability, reimbursement pathways, regulatory environments, and clinical trial participation within a single access-focused framework may provide a more comprehensive understanding of global outcome gaps in advanced NSCLC.

The aim of this narrative review is to synthesize current evidence on disparities in access to diagnostics and systemic therapies for advanced NSCLC across high-, middle-, and low-income settings. We focus on structural, economic, and regulatory determinants that shape implementation of modern diagnostic workflows, availability of novel treatments, and participation in clinical trials, with the objective of clarifying where inequities arise and how they influence global outcomes.

## Methods

This review was conducted as a narrative review of the published literature on global disparities in access to diagnostics and systemic therapies for advanced NSCLC. The search covered publications from 2010 to 2025, a period selected to capture the modern era of molecularly guided NSCLC care from the clinical establishment of targeted therapy through the immunotherapy era. Literature was identified through targeted searches of PubMed using combinations of disease- and domain-specific search terms including ‘non-small cell lung cancer’, ‘NSCLC’, ‘disparities’, ‘access’, ‘molecular testing’, ‘diagnostics’, ‘treatment access’, ‘reimbursement’, ‘low- and middle-income countries’, and country- or region-specific terms. Additional publications were identified through review of reference lists of included articles and key guidelines, and through purposive retrieval of reports and recommendations from international health organizations and professional bodies. Searches were conducted in PubMed.

Inclusion criteria were applied as follows: publications were included if they addressed disparities in access to diagnostic technologies or systemic therapies for advanced NSCLC; reported real-world data, health system analyses, or policy-relevant findings applicable to clinical implementation; or represented international clinical guidelines or consensus recommendations from recognized professional bodies. To mitigate regional bias, literature from high-, middle-, and low-income settings was actively prioritized, including the JTO regional lung cancer series, which provided expert-authored, country-specific perspectives on lung cancer epidemiology, diagnostics, and treatment access across multiple geographic regions. Exclusion criteria comprised publications focused exclusively on treatment efficacy or molecular biology without addressing access, availability, or health system context, and studies without applicability to real-world care delivery across income settings. Preference was given to peer-reviewed publications, multi-institutional datasets, and registry-based analyses over single-institution retrospective series where alternatives were available. The evidence was synthesized thematically across income settings, with findings triangulated where possible across multiple independent regional sources to reduce dependence on single studies.

### Disparities in access to diagnostics

#### Clinical diagnosis

Accurate staging is a prerequisite for effective management of advanced lung cancer. In high-income settings, this is achieved through multimodal imaging standard integrating computed tomography (CT) for initial assessment, positron emission tomography-CT (PET-CT) for detection of nodal and distant metastases, and magnetic resonance imaging (MRI) of the brain for evaluation of intracranial disease – together enabling high diagnostic sensitivity and precision staging that directly informs treatment eligibility and decision-making (13, 14). Outside high-income settings, access to even individual components of this imaging standard is far from guaranteed.

Global data from the International Atomic Energy Agency (IAEA) illustrate the scale of this gap: PET-CT density ranges from 3.43 scanners per million population in HICs to just 0.01 per million in LICs, with upper-middle-income countries (UMICs) occupying an intermediate position at 0.36 scanners per million. MRI availability follows a similar gradient, from 27.10 per million in HICs to 0.18 per million in LICs (15, 16). As a result, most LMICs and LICs rely predominantly on conventional imaging (e.g. X-Ray), leading to incomplete or delayed staging that directly affects treatment eligibility and outcomes.

Structural health-system limitations compound these equipment gaps. In many LMICs and LICs, comprehensive cancer centers are concentrated in a small number of urban hubs, leading to prolonged referral chains and substantial travel distances for patients – though systematic, income-stratified data on cancer center density remain scarce, limiting direct global comparisons. As an illustrative example, Vietnam has only five comprehensive cancer centers serving a population of approximately 100 million (0.05 centers per million inhabitants) (17), and comparable limitations have been reported across SSA, where fewer than 100 hospitals provide any form of cancer care for a population exceeding 1.5 billion (18). This geographic bottleneck is inseparable from a profound workforce deficit. A global survey of the clinical oncology workforce across 93 countries found that in 42% of countries a single oncologist is responsible for more than 500 new cancer cases per year, and in 29% of countries — predominantly in Africa and Asia — this burden exceeds 1,000 new cases per oncologist annually. Eight countries had no clinical oncologist available at all. In stark contrast, in 24% of countries — predominantly high-income — one oncologist serves fewer than 150 new patients per year. The mortality-to-incidence ratio, a proxy for the quality and timeliness of cancer care, exceeded 70% in 66% of African countries surveyed, compared to none in Europe or the Americas (19).

Diagnostic delays are well documented across LMICs. In a cohort of 331 high-risk patients in Western Kenya, the mean symptom duration prior to diagnosis was eight months, and more than half of patients had visited between seven and ten hospitals before lung cancer was confirmed (20). In India, delays exceeding three months between symptom onset and treatment initiation have been reported (21), while a Brazilian retrospective analysis of nearly 3,000 patients with metastatic NSCLC found that only 10.2% presented with a Karnofsky performance status ≥90% at referral to oncology services, consistent with late-stage presentation driven in part by delayed diagnostic pathways (22, 23).

#### Pathological and molecular diagnosis

Modern lung cancer pathology requires both accurate histological subtyping and comprehensive molecular profiling. In patients with advanced NSCLC, current international guidelines recommend testing for a broad panel of oncogenic driver alterations — including *EGFR*, *ALK*, *ROS1*, *BRAF*, *MET*, *RAS*, *HER2*, *RET*, *NTRK*, and *NRG1* — as well as PD-L1 expression (24–27). This recommendation reflects a rapid diagnostic evolution in HICs: where single-gene PCR-based assays once sufficed, NGS has emerged as the preferred method, enabling simultaneous interrogation of multiple biomarkers from small biopsy or cytology specimens (28, 29). Building on this, contemporary HIC practice increasingly employs comprehensive genomic profiling (CGP) through broad pan-cancer panels such as the Illumina TruSight Oncology 500 (TSO500), which consolidates hundreds of genes into a single test and reports multiple variant types and signatures (30). At the leading edge, whole-exome sequencing (WES) and whole-genome sequencing (WGS) are entering select clinical pathways, with recent ESMO recommendations acknowledging their progressive incorporation into practice. The field is also entering an AI-enabled phase in which computational pathology, radiomics and multi-omics models surface new predictive signatures beyond conventional PD-L1 or single variants (31).

Building on these diagnostic advances, high-income systems have increasingly institutionalized molecular tumor boards (MTBs) to navigate the growing volume of biomarkers and therapy options. National models include France’s INCa network of 28 regional molecular genetics centers, which mainstreamed high-throughput sequencing and case discussions at scale for patients with soft-tissue carcinoma or colorectal carcinoma (32). Germany’s national Network Genomic Medicine (nNGM) Lung Cancer links more than 30 certified centers and hundreds of partner sites, coupling multiplex testing with standardized treatment recommendations and demonstrating outcome benefits in real-world analyses (33). The UK NHS (National Health Service) Genomic Medicine Service embeds genomic multidisciplinary team meeting (MTDs) and MTBs across Genomic Laboratory Hubs to deliver uniform interpretation and access (34). Japan went a step further in 2019, making CGP testing reimbursable only in government-designated hospitals that convene MTBs, with a national data center (C-CAT) supporting interpretation and feedback loops (35, 36). At the pan-European level, the ESMO Precision Oncology Working Group has issued NGS recommendations (2024) and dedicated guidance (2025) on how MTBs should be constituted and audited—membership, workflows, documentation, and quality indicators—to harmonize practice across countries (37, 38). In parallel, ESMO recommendations on clinical reporting of genomic testing now call for structured pathology reports that specify assay type (e.g., panel/NGS), genes/regions covered, analytic performance (coverage/read-depth), variant details (Human Genome Variation Society (HGVS) nomenclature, Variant Allele Frequency (VAF)), oncogenicity and clinical actionability (e.g., ESMO Scale for Clinical Actionability of molecular Targets (ESCAT) tiering), and clear, implementable conclusions for the MTB/clinician—standards that help translate complex profiles into consistent decisions (39). These structured frameworks represent the current standard against which global disparities must be measured.

In strong contrast, LICs and LMICs lag behind in pathology progress and still face substantial barriers in implementing diagnostic workflows. In many parts of SSA, diagnosis continues to rely on cytology specimens such as sputum smears or pleural effusion samples, owing to limited availability of image-guided biopsies and interventional pulmonology (40, 41). These methods may confirm malignancy but frequently do not allow histological subtyping or preserve sufficient tissue for immunohistochemistry and molecular testing.

The rollout of PD-L1 immunohistochemistry and molecular testing is highly heterogeneous across these settings. Southeast Asian countries such as Thailand and Vietnam have introduced in-house *EGFR*, *ALK*, and PD-L1 testing in tertiary institutions, but coverage remains uneven in provincial hospitals, and NGS is largely limited to research collaborations or outsourced laboratories (17, 42). In much of Africa, PD-L1 and molecular assays are either nonexistent or available only via outsourced partnerships, with turnaround times of several weeks due to sample transfer logistics (43, 44). Outsourcing introduces delays and cost barriers that frequently render results clinically outdated by the time they are returned. Reimbursement and funding pose additional obstacles: in many low-income countries, molecular testing is entirely out-of-pocket, with costs far exceeding average monthly incomes. Even in some middle-income contexts, tests like *EGFR* or *ALK* may be routinely performed but reimbursement remains fragmented and inconsistent.

Finally, workforce shortages and infrastructure deficits exacerbate challenges. An informal survey conducted in 2012 revealed that most African countries averages fewer than one pathologist per million population, leading to long delays in slide review and frequent misclassification (45). Advanced molecular training is limited, and many pathologists lack experience with NGS or PD-L1 interpretation. Lab capacity is often constrained by reagent stock-outs and infrastructure gaps that disrupt even routine histology.

### Disparities in access to novel treatment options

Over the past two decades, the therapeutic armamentarium for advanced NSCLC has expanded at speed, with successive generations of targeted therapies (*EGFR, ALK, ROS1, BRAF, MET, RET, NTRK, KRAS* G12C, *HER2*) and a crowded PD-1/PD-L1 class with various approvals and a vast number of “me-too” drugs. In HICs, access pathways are designed to translate these approvals rapidly into practice.

Germany, for example, permits immediate market entry at the manufacturer’s price upon EMA authorization, with the AMNOG (Act on the Market Reorganization of Medicinal Products) process to set the national price running in parallel; since 2023 the negotiated price applies retroactively from month seven, but prescribability directly after EMA approval remains intact, delivering one of the fastest accesses to new treatment options worldwide (46–48). In most European countries, newly approved drugs by the EMA cannot automatically be prescribed but require a clinical benefit and price assessment prior their national reimbursement listing. However, some countries have established robust Early Access (EA) routes. In France, a recent exchange of the former ATU (“Autorisation Temporaire d’Utilisation”) by the new “Accès précoce” (AAP/AAC) framework allows companies to request Early Access Authorization – before and after EMA approval – while the HAS (“Haute Autorité de Santé”) evaluates the clinical benefit and the CEPS (“Comité économique des produits de santé”) negotiates on pricing for formal reimbursement (49). EA indications are mostly approved within 70 days (with a maximum duration of 90 days) with a high HAS approval rate of 80% (50). Similarly, in the UK, the NHS Cancer Drug Funds provides managed-access to promising new oncological drugs as a bridging mechanism. Meanwhile, the NHS closely collaborates with the National Institute for Health and Care Excellence (NICE) to collect more data for the Technology Appraisal (TA) guidance recommendations after which the NHS is legally obliged to fund a treatment (51). In Spain, a national Therapeutic Positioning Report (IPT) is prepared after EMA approval and submitted to the CIPM (“Comisión Interministerial de Precios de los Medicamentos y Productos Sanitarios) which undertakes the price and reimbursement decision. However, a review by the Spanish Lung Cancer Group states that, compared to other European countries, Spain has the lowest access to EMA-approved cancer drugs with an average delay of more than 600 days to national listing (52). Even though EMA-approved drugs for common alterations such as *EGFR, ALK, ROS1* and *BRAF V600* are widely available in all European countries, newer therapies for biomarkers like *RET, NTRK, MET exon 14 skipping*, *KRAS G12C* and *NRG1* are inconsistently accessible, being standard-of-care in most, but not all countries (53).

In the United States, data indicate that drugs account for 20% of total cancer care with an increasing proportion over time. In 1995, insurers paid $54,100 in drug costs for a year of life. In 2013, the sum already increased to $207,000, accelerating much faster than other components of cancer care (54). After FDA approval, new oncology drugs are almost immediately available, giving patients rapid access to cutting-edge therapies. However, this speed comes at a cost: launch prices for cancer drugs are far higher in the US than in other HICs, since the manufacturers are permitted to set the prices as high as the market will bear and increase the costs over the course of time – even in absence of new evidence of clinical benefit or changes in use (55). As an instance, prices in the US are two to three times higher than in other peer countries (56). In 2023, launch prices exceeded $100,000 per year for 95% of new anticancer therapies (57, 58). Public insurance covers nearly all oncology drugs but patients historically face high “out-of-pocket” (OOP) burdens, leading to financial debt and potential nonadherence and early discontinuation of treatment with the risk of poor prognosis and lower overall survival rates. As a consequence, governmental efforts have been made to decrease costs by introducing parity and price transparency laws. Substantial changes were implemented by the Inflation Reduction Act (IRA) in 2022, requiring the Secretary of Health and Human Services (HHS) to directly negotiate prices with manufacturers for therapies that are covered by Medicare (federal insurance for people ≥65 years or with certain disabilities) Part B (infusion/injection drugs given in hospitals) or Part D (oral drugs delivered through private plans that create formularies) (59). Furthermore, if companies raise prices faster than the inflation, they must pay rebates to Medicare. Additionally, OOP costs in Part D are capped at $2,000 per year (55). Negotiations are expected to save Medicare $98.5 million over the next years, however manufacturer revenues could result in fewer new drugs entering the market during the same period (60).

Other HICs, like Canada, Australia, Japan and South Korea, follow similar procedures to Europe which include multi-step pathways, evaluating the clinical efficacy and cost-effectiveness, followed by price negotiations and only then the formal listing on the national reimbursing list (61–64).

The situation in lower-income settings is highly heterogeneous. The World Health Organization (WHO) Essential Medicines List (EML) serves as a global reference for medicines considered fundamental to meet priority health needs, based on evidence of efficacy, safety, and cost-effectiveness. For oncology, the EML has traditionally included widely available cytotoxic and hormone therapies, but more recent editions have also incorporated high-cost targeted agents (e.g. TKIs) and immunotherapies, reflecting their proven clinical benefit despite affordability challenges (**Table 1**) (65). National Essential Medicines Lists (NEMLs) are country-specific adaptations of the WHO EML that guide procurement and reimbursement within national health systems (66). While alignment with the WHO EML is encouraged, discrepancies frequently occur: newer targeted therapies may be excluded from NEMLs due to high costs, patent restrictions, limited diagnostic infrastructure, or local epidemiologic priorities. Additionally, some NEMLs may lag behind the latest WHO recommendations if they are not regularly updated. As a result, access to essential lung cancer drugs can vary substantially across countries.

**Table 1:** Global world health essential medicines list (WHO EML) for NSCLC.

WHO model list of essential medicines for NSCLC 24th list (2025)	
Afatinib	First added in 2019
Atezolizumab	Application rejected in 2019 and 2023, first added in 2025 as therapeutic alternative to pembrolizumab
Carboplatin	First added in 2009
Cemiplimab	Application rejected in 2023, first added in 2025 as therapeutic alternative to pembrolizumab
Cisplatin	First added in 1984
Erlotinib	Application rejected in 2017, first added in 2019 as therapeutic alternative to afatinib
Etoposide	First added in 2015
Gefitinib	First added in 2019 as therapeutic alternative to afatinib
Gemcitabine	First added in 2015
Paclitaxel	First added in 2015
Pembrolizumab	Application rejected in 2019, first added in 2025
Vinorelbine	First added in 2015

In China, an upper-middle-income country, oncology drugs are reviewed and authorized by the National Medical Products Administration (NMPA), which since major reforms beginning in 2015 has introduced priority review, breakthrough therapy, and conditional approval pathways, accepted more foreign clinical trial data, and substantially reduced the historic “drug-lag” for innovative agents, bringing access timelines closer to the U.S. and European Union (67). Once on the market, broad public coverage is dependent on the inclusion in the National Reimbursement Drug List (NRDL), negotiated annually by the National Healthcare Security Administration. NRDL negotiations typically require large price cuts at listing—recent cycles report average discounts around 50% (68). These negotiations have measurably reshaped spending and use: analyses across 2017–2024 show sharp price reductions at NRDL entry and subsequent volume surges, notably among PD-1/PD-L1 inhibitors, where domestic agents gained rapid uptake following steep cuts (68, 69). A parallel pillar of China’s approach has been domestic innovation: a wave of homegrown immunotherapies (e.g., camrelizumab, sintilimab, tislelizumab, toripalimab) and targeted drugs (e.g. savolitinib, taletrectinib, sunvozertinib) has expanded therapeutic choice and intensified price competition, with pharmacoeconomic studies increasingly finding domestic PD-1 regimens cost-effective at negotiated listing prices (70). Overall, the combination of accelerated regulatory pathways, centralized NRDL price-volume bargains, and a maturing local biopharma sector has moved China from slow, high-price access towards faster availability at materially lower reimbursed prices, even as OOP burdens and diagnostic inequities still produce regional variability in real-world access.

In other Asian UMICs, access remains more limited. In Thailand, for example, all FDA-approved drugs are also approved by the Thai FDA; however, the delay between U.S. FDA and Thai FDA approvals often spans from two to three years, and reimbursement is highly restricted. Under the Civil Servant Medical Benefit Scheme (CSMBS), patients can access targeted therapies and immunotherapies (e.g. gefitinib, osimertinib, ceritinib, atezolizumab), but those covered by the Social Security Scheme (SSS) and Universal Coverage Scheme (UC)—together encompassing over 60 million people—lack reimbursement for TKIs and immunotherapy, leaving most patients dependent on OOP financing. The Oncology Prior Authorization Program (OCPA) provides a pathway for high-cost drugs in the CSMBS, but no such mechanism exists for UC or SSS beneficiaries. Moreover, molecular testing is neither routine nor reimbursed under UC/SSS, and NGS remains largely confined to private hospitals or research projects, reinforcing disparities in access to precision oncology. Pharmaceutical companies have occasionally provided free testing programs, but these are limited in scope (42).

In Latin America, the Latin American Consortium for the Investigation of Lung Cancer (CLICaP) was formed in 2011 and includes more than 75 lung cancer researchers from most Latin American Countries and offers a series of training courses in the field of thoracic oncology to enhance education on new treatments as well as to strengthen research ambitions in the area (71). In Brazil, for example, most first and second generation TKI are available. However, national approvals can be challenging and delayed, compared to FDA and EMA approvals. As an instance, the clinical use of crizotinib for *ALK*-rearranged NSCLC was only approved in 2016, multiple years later than in the US and Europe and it has been estimated that this caused more than 700 premature deaths in Brazil (23). In Mexico, for example, patients being treated at reference institutions all undergo molecular testing, and first-generation TKIs (erlotinib, gefitinib, crizotinib) are fully reimbursed by public insurance. However, only 7% of the population are covered by private insurance that might cover next-generation TKIs, leaving 93% of the Mexican population excluded to more efficacious and less toxic treatments. Despite wide availability of early-generation TKIs, only 50% of all *EGFR*-positive and 30% of all *ALK*-positive NSCLC patients receive targeted treatment (72). Similarly, in Colombia, where a variety of personalized medicines are named in the national recommendations of the Ministry of Health, including third-generation TKIs as osimertinib or other targeted therapies for rare alterations like *ROS1* (crizotinib) and *NTRK* (larotrectinib), it is estimated that only 50–60% of the population – usually treated in institutions located in main Colombian cities – has access to molecular profiling which mainly consists of *EGFR*, *ALK* and increasingly for *ROS1* (73). Due to significant price differences between early- and later-generation TKIs and low success on importing “available-vital medicine” for individual treatment strategies, comprehensive molecular profiling remains of low interest (73).

In LMICs, lung cancer care is highly dependent on the country. In India, for example, most targeted agents (including generics for early generation TKIs) and immunotherapies are available. However, public insurances mostly only cover cytotoxic therapy and access remains limited due to low insurance rates of approximately 30% of the whole population. Recent governmental initiatives, like Ayushman Bharat Pradhan Mantri Jan Arogya Yojana, aim to improve coverage. Nevertheless, most patients face substantial financial barriers due to the need of OOP payments (21). Other countries, like Pakistan, even developed institutional guidelines using a cost-benefit approach to mostly focus treatment on younger and potentially curable patients using available generic chemotherapy and radiotherapy while allocating most of older patients and patients with advanced cancer to palliative therapy alone (74).

Notably, many LICs cannot systematically deliver even baseline components of precision NSCLC care. Multiple reviews document that oncology drug access is constrained by price, supply chains, workforce and diagnostic capacity, leading to reliance on platinum doublets and older cytotoxics, with sporadic access to TKIs and minimal immunotherapy penetration.

In parts of SSA, the number of cancer treatment centers relative to population remains small and budgets for high-cost medicines are limited, compounding delays and attrition along the treatment pathway. Even where NEMLs have begun to list modern NSCLC agents (e.g., Kenya now lists pemetrexed, bevacizumab and even osimertinib in defined settings) (75), formulary listing does not guarantee routine availability, financing, or trained staff to use them. An analysis by Olatunji et al. comparing the WHO EML of 2019 and the NEMLs of Keyna (2019), Uganda (2016) and Rwanda (2015) showed an alignment of 93.4%, 70.5% and 41.1%, respectively. Furthermore, they assessed affordability for the government as well as for the patients themselves (OOP) and defined affordability as followed: treatment is affordable for the government if the annual cost is lower than three times the Gross National Income (GNI) and for OOP payment, a 30-day treatment period is affordable if the cost is lower than 1 day’s minimum wage. They concluded that most standard chemotherapy regimens (mostly generics) are affordable to governments but not to patients as OOP. However, newer therapies like targeted treatment and immunotherapy remain unaffordable, even for governments (76). Consequently, only a scarcity of hospitals can make use of these treatments. As an instance, a survey that was conducted in almost 30 countries in SSA revealed that only 46% of the medical staff, mostly physicians, were familiar with immunotherapy and 11% indicated being adequately trained to administer it, of which only 52% felt confident in the management of immune-related adverse events (77). Consequently, the National Comprehensive Cancer Network (NCCN) adapted international guidelines for the Middle East and North Africa (MENA) and SSA region (78–80). Additionally, the Multi-National Lung Cancer Control Program (MLCCP) was formed by various countries in the region (Kenya, Tanzania, Swaziland and South Africa) to further enhance quality of care by fostering scientific exchange and generate joint data (20). Some countries already implemented cancer registries, like the South African National Cancer Registry, a pathology-based system, that was established in 1986 and last updated in 2014, aiming to provide statistics on incidence of lung cancer (81).

Across these diverse settings, several recurring patterns are apparent: regulatory approval and reimbursement remain structurally decoupled in most countries, access is frequently stratified within countries by insurance status or ability to pay regardless of formal approval, therapies for rarer biomarker-defined subgroups remain largely confined to high-income settings, and drug pricing anchored in HIC markets renders newer agents unaffordable at the government or patient level across much of the world.

### Disparities in access to clinical trials

Clinical trials are a cornerstone of progress in thoracic oncology, serving both as engines of innovation and access pathways to novel therapies before regulatory and reimbursement approval. Since the 2000s, the number of oncology studies initiated worldwide has risen markedly. An analysis of *clinicaltrials.gov* reported 638 studies in 2000 compared to 6571 in 2021 (82).

**Figure 1:**
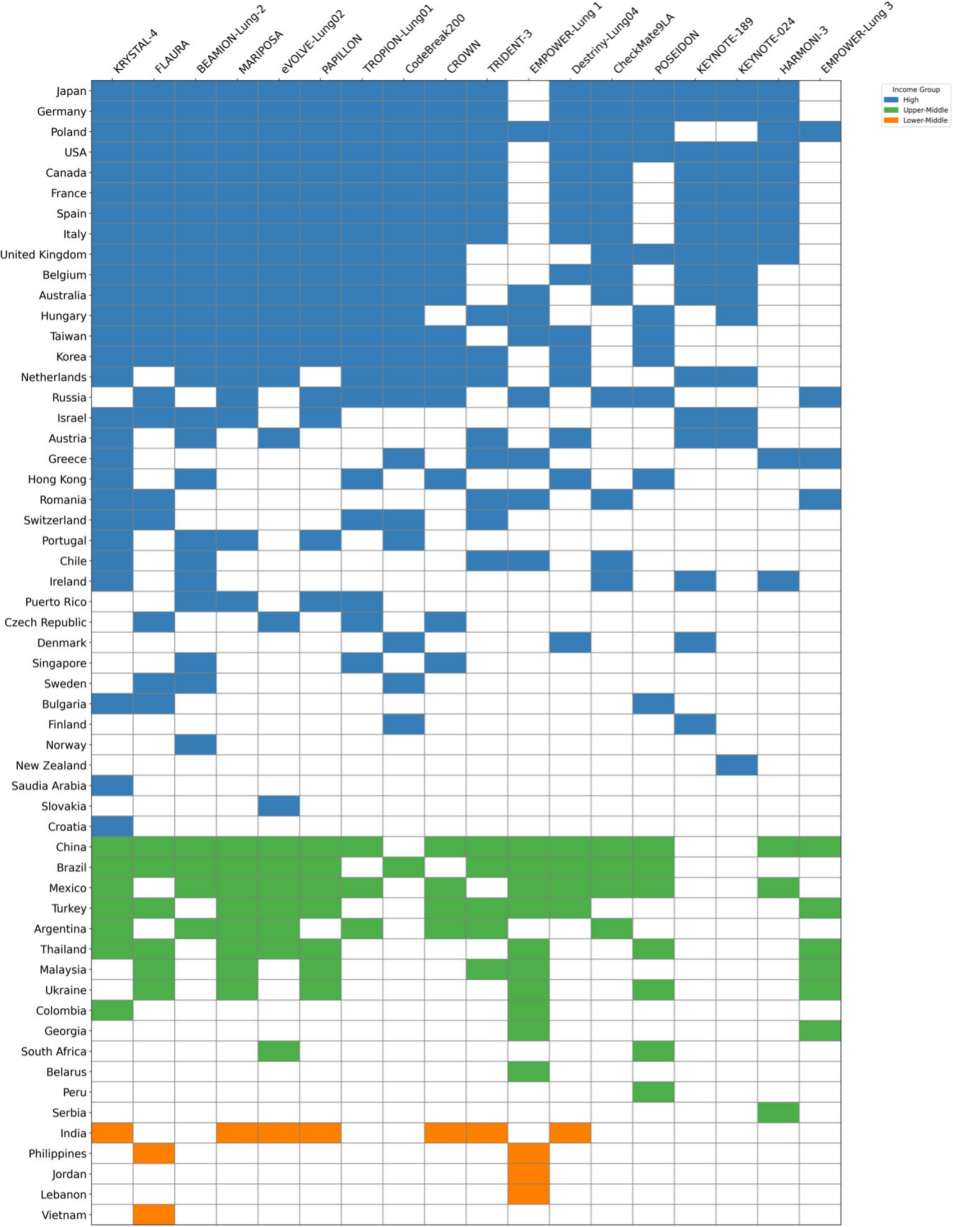
Overview of major phase III trials in advanced NSCLC. Countries are shown on the y-axis and individual trials on the x-axis. Colored squares indicate participation of a given country in the respective trial; absence of square indicates no participation. Countries are color-grouped by the World Bank income category to highlight disparities in global trial representation.

Regulatory frameworks in HICs, together with strong academic–industry partnerships, ensure that the vast majority of global phase II–III registrational NSCLC trials are conducted in these regions (**Figure 1**). While innovative drugs are likely to become widely accessible to patients in HICs over time, clinical trials can provide access years before formal approval, depending on the country. As stated before, the mean time from regulatory to reimbursement approval can vary substantially, ranging from 128 days (Germany) to 629 days (Spain) in Europe. Between 2018 and 2021, 98% of all new EMA-approved lung cancer therapies were fully available in Germany, compared with 35% in the UK and 28% in Spain (52) A considerable proportion of patients in HICs thus benefit from participation in trials of innovative drugs that might otherwise only become accessible to them after years – if ever. Although patients in HICs generally benefit from robust trial infrastructure, comprehensive diagnostics, and efficient referral networks, only a minority are ultimately enrolled. A retrospective evaluation at US trial centers showed that while nearly 23% of patients were eligible for a clinical trial, ultimately only about 8% were enrolled (83).

**Figure 2:**
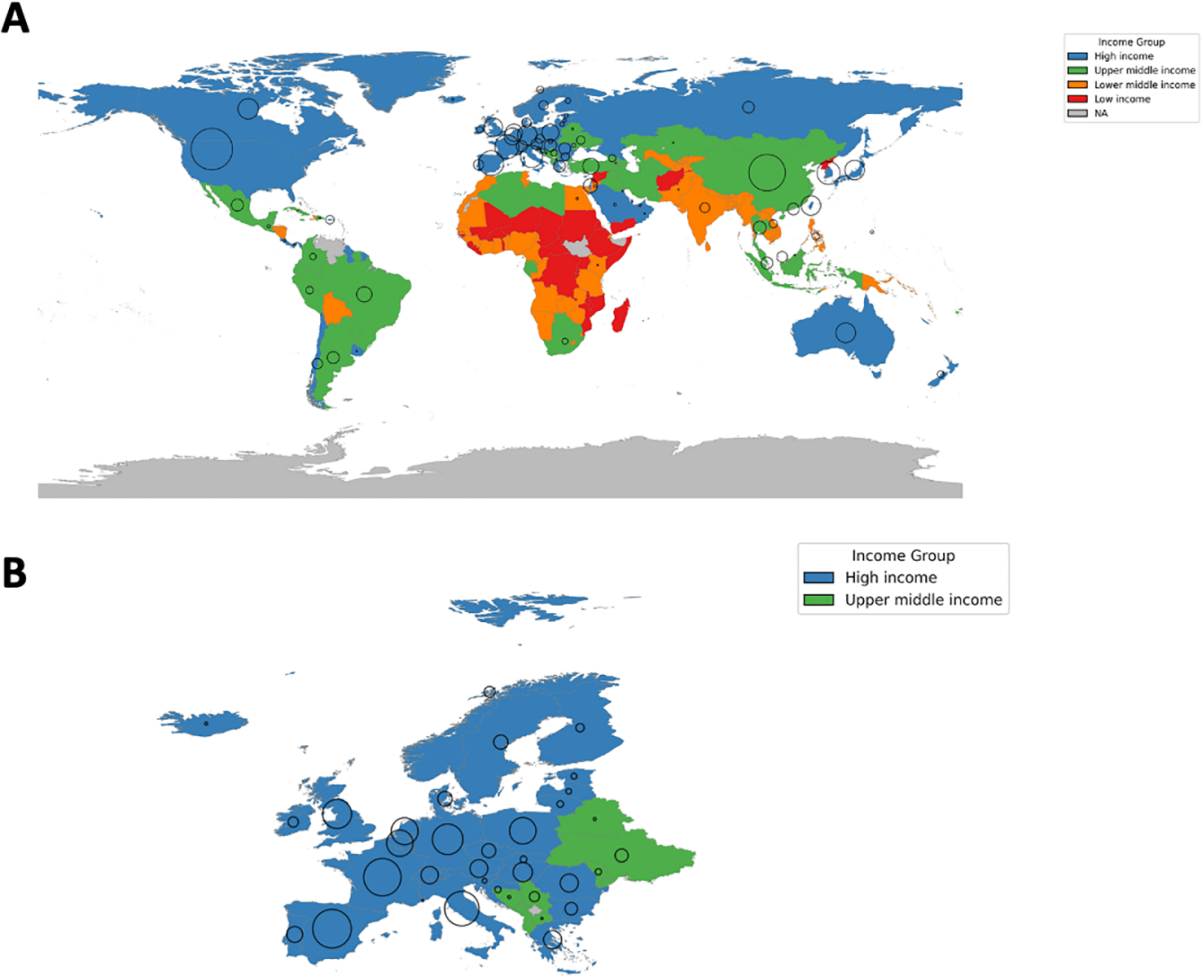
Clinical trial access on a global and european level. Proportional bubble map illustrating the geographic distribution of active clinical trials in NSCLC as listed on *ClinicalTrials.gov* accessed in October 2025 (status: “Active, Not Recruiting” and “Recruiting”). The countries are color-coded by World Bank Income group; the bubble size reflects the absolute number of registered trials per country, with larger bubbles representing greater trial activity. Trial counts were not adjusted for population size or disease incidence, as the figure is intended to illustrate the geographic concentration of clinical research activity rather than per-capita trial availability. Normalization would obscure the marked clustering of trials in a small number of high-income countries that dominate global trial conduct. **(A)** Global Distribution of active NSCLC trials; **(B)** European Distribution of active NSCLC trials.

While research activity and study density remain highest in HICs, clinical trials have become increasingly globalized in recent years, with a growing shift toward middle-income settings (84). (**Figure 2**) More than 80% of phase III studies involving immunotherapy in NSCLC were conducted at least in part in UMICs and LMICs. China has been the leading country for phase II/III trials since 2015 and for phase I trials since 2023 (82). In Latin America, where many countries are classified as MICs, the number of lung cancer studies also increased significantly between 2001–2011 and 2012–2021 (100 vs. 173) (85).

By contrast, LICs remain almost completely excluded; during the same period, not a single phase I–III NSCLC trial was registered in LICs. Moreover, despite this apparent shift, true globalization remains limited: 85% of all cancer trials (including lung cancer) conducted between 1999 and 2022 were national, and only 6% involved collaborations between HICs and MICs/LICs (86). Driven by strong population growth, particularly in SSA, cancer will play an increasingly important role in LICs in the coming decades, yet these countries remain markedly underrepresented in clinical trials (87).

## Discussion

Global inequities in lung cancer care remain profound, reflecting disparities across the entire continuum of diagnosis, treatment, and access to innovation. While patients in HICs benefit from comprehensive molecular profiling, access to multidisciplinary expertise, and rapid uptake of novel targeted and immune therapies, patients in MICs and LICs often rely on histologically confirmed diagnosis with limited immunohistochemical and molecular testing. These gaps are particularly striking in regions, where the prevalence of oncogene-addicted disease might be underestimated but highly probable due to environmental exposure (e.g. wood smoke exposure likely causing *EGFR*-mutated NSCLC), and where the availability of less toxic oral therapies could have a disproportionate benefit, given the long travel distances many patients face and the challenges of managing toxicity outside major centers.

Closing these gaps requires not only technical solutions but also systemic and social strategies. Establishing basic cancer registries is fundamental to generating reliable data on incidence, biomarker prevalence, and outcomes—information that is a prerequisite for rational planning. Pharmaceutical industry funding, coupled with international collaboration, can help scale access to diagnostics and therapies in underserved settings. At the same time, empowering advocacy groups—particularly when supported by long-standing organizations such as the IASLC—can provide the social and political momentum needed to push governments, industry, and societies toward equitable solutions.

Pricing strategies represent a further barrier. Drug prices are often set not on demonstrated clinical value but on the appeal of addressing an unmet need, with external reference pricing spreading high launch prices across international markets. This practice risks replicating inflated benchmarks even in countries with limited resources. A value-based pricing approach, aligned with health outcomes and societal benefit, is essential to ensure that life-extending therapies become available to the largest possible number of patients worldwide.

A feasible path forward is to operationalize equity through implementable packages: (I) a minimum diagnostic standard (CT-based staging, quality-assured histology with small IHC panels, and at least *EGFR/ALK*/PD-L1 testing with defined turnaround targets), (II) scalable molecular networks (regional hubs, specimen transport, external quality assurance, and virtual MTB support), (III) an access package for medicines (EML-aligned procurement, differential pricing, pooled purchasing, and rapid uptake of quality-assured generics/biosimilars), and (IV) trial and evidence strategies that include LMIC/LIC-relevant endpoints (time-to-treatment, access barriers, affordability, and real-world implementation). Translating these packages into practice will require coordinated action across multiple actors and governance levels. For packages I and II, established international oncology bodies have already developed applicable frameworks: the NCCN has produced resource-stratified guideline adaptations — organized into Basic, Core, and Enhanced resource tiers — that have been implemented in sub-Saharan Africa and the Middle East and North Africa region (78–80), and the IASLC has systematically documented disparities in biomarker testing access across income settings and has committed to targeted initiatives addressing awareness, access, processes, and policy improvements globally, as demonstrated by its recurring global biomarker surveys conducted across 90 countries (88). For package III, relevant structural precedents exist in other disease areas. Gavi, the Vaccine Alliance — a public-private partnership that pools procurement demand across low-income countries to negotiate reduced prices and uses advance market commitments to incentivize manufacturer supply — and the Global Fund to Fight AIDS, Tuberculosis and Malaria — a multilateral financing mechanism that aggregates purchasing volumes to secure quality-assured medicines at negotiated prices — have demonstrated that coordinating industry engagement with WHO oversight and national health ministry participation can substantially reduce commodity costs and expand access in LMICs and represent transferable models for oncology drug access (89, 90). For package IV, embedding LMIC and LIC sites into registrational trial designs will require regulatory incentives from the FDA and EMA, as well as dedicated funding streams from national cancer institutes or organizations such as the Union for International Cancer Control (UICC). Aligning guideline recommendations with resource-stratified pathways and embedding implementation science into thoracic oncology research will be critical to translate discovery into delivery at global scale.

Several limitations of this review warrant acknowledgment. As a narrative rather than systematic review, the literature search was not exhaustive; searches were restricted to PubMed and additional databases were not systematically consulted, and the selection of evidence was necessarily guided by relevance and regional representativeness rather than a predefined protocol. Furthermore, granular data on molecular testing rates, drug availability, and real-world treatment access remain scarce in many LMICs and LICs — particularly outside major academic centers — and are often derived from single-institution reports, expert surveys, or indirect proxy measures rather than population-based sources. This data scarcity means that the present review does not claim completeness, and the true extent of disparities in underrepresented regions is likely greater than the available evidence allows us to capture. Studies from LICs and LMICs more broadly remain underrepresented in the published literature, which may further contribute to an underestimation of existing inequities. **Figure 1** and **2** illustrate the geographic distribution of clinical trial activity in absolute terms and were intentionally not normalized for population size or cancer incidence, as normalization would risk obscuring the marked absolute concentration of trials in a small number of high-income countries. However, it should be acknowledged that absolute trial counts alone do not capture per-capita trial availability, and countries with large populations and high cancer incidence — such as China or India — may appear overrepresented relative to their per-capita access. Readers should therefore interpret the figures as illustrating geographic clustering of research activity rather than equitable or per-capita trial access. Finally, the rapidly evolving landscape of both diagnostic technologies and drug approvals means that some specific data points referenced in this review may have been superseded by subsequent developments.
